# The Effect of Temperature and Storage Duration on the Quality and Attributes of the Breast Meat of Hens after Their Laying Periods

**DOI:** 10.3390/foods12234340

**Published:** 2023-12-01

**Authors:** Anna Augustyńska-Prejsnar, Paweł Hanus, Małgorzata Ormian, Miroslava Kačániová, Zofia Sokołowicz, Jadwiga Topczewska

**Affiliations:** 1 Department of Animal Production and Poultry Products Evaluation, Institute of Food and Nutrition Technology, University of Rzeszow, 35-959 Rzeszow, Poland; aaugustynska@ur.edu.pl (A.A.-P.); mormian@ur.edu.pl (M.O.); zsokolowicz@ur.edu.pl (Z.S.); 2 Department of Food Technology and Human Nutrition, Institute of Food and Nutrition Technology, University of Rzeszow, 35-959 Rzeszow, Poland; phanus@ur.edu.pl; 3 Institute of Horticulture, Faculty of Horticulture and Landscape Engineering, Slovak University of Agriculture, 949 76 Nitra, Slovakia; miroslava.kacaniova@gmail.com

**Keywords:** post-laying hen, storage time, storage temperature, MALDI-TOF MS, physical and chemical characteristics, sensory quality

## Abstract

The purpose of this study was to evaluate the effect of temperature (2 °C and 6 °C) and storage duration on the quality and attributes of hens’ breast meat after their laying periods. The study included physicochemical characteristics (pH, drip loss, colour, shear force), microbiological quality (total Enterobacteriaceae family and Pseudomonas count), and sensory quality. Bacterial identification was performed using matrix-assisted laser desorption/ionisation time-of-flight mass spectrometry. The increased meat pH and drip loss was greater at 6 than 2 °C (*p* < 0.05). An increase in the tenderness of the meat stored at 6 °C was found as early as day 4, as well as at 2 °C on day 8 of storage (*p* < 0.05). On day 4 of storage, the meat was characterised by a darker colour than on the first day, but the darkening was greater at 6 °C than at 2 °C (*p* < 0.05). At 6 °C, on day 4 of storage, there was an increase in yellow saturation (b*) of the meat, which was higher at 6 °C than at 2 °C (*p* < 0.05). At 2 °C, the total bacterial count and number of *Pseudomonas* spp. in the meat gradually increased along with increasing storage duration, reaching 4.64 log cfu/g and 4.48 log cfu/g, respectively, on the 8th day of storage. At 6 °C, on the sixth day of storage, the total bacterial count in the meat exceeded 7 log cfu/g, considered the limit of microbiological safety. The meat stored at 2 °C had an acceptable sensory quality until the 8th day of storage. The study shows that storage at 2 °C preserves the sensory characteristics and microbiological safety of the hen meat longer at an acceptable level after the laying period. Extended storage life may be of importance to consumers and the meat industry.

## 1. Introduction

Laying-hens’ meat, after termination of the egg-laying period, is most often disposed of or used in animal fodder [1]. The use of meat from poultry production after the end of the laying period for consumer purposes is marginal, mainly due to its less favourable technological and sensory characteristics [2]. However, the increased demand for local and regional food products observed in recent years offers new opportunities and perspectives for using the meat of native and local breeds, e.g., RIR hens of free-range/organic breeding [3]. At the same time, it should be emphasised that the use of such raw material is part of the need to reduce food waste. According to the concept contained in the European Green Deal, a 10% reduction in food processing waste should be sought by 2030 [4,5]. The most common method of preserving poultry meat for short-term storage is refrigeration. This, however, requires periodic storage of larger amounts of hen meat. A significant number of studies were conducted concerning the effects of time and storage conditions on the quality of broiler chicken meat [6,7,8,9,10]. However, there is a lack of research concerning the influence of time and storage conditions on the quality of hen meat after termination of the laying period, as well as a lack of estimates on the length of its shelf life. Shelf life is the recommended maximum storage time allowing for safe food consumption. Poultry meat is perishable and a loss/drop in quality occurs quickly during its storage [11,12]. Inappropriate storage conditions result in the development of microorganisms and enzymatic changes that cause meat spoilage [13,14,15,16,17,18], deterioration of its sensory characteristics [12,19,20] and physicochemical characteristics [21]. In order to delay spoilage and lengthen the shelf life, poultry meat is most often stored in cool conditions, at a temperature of 0 °C to 7 °C [22,23,24]. Storage in cool conditions limits the development of microorganisms and slows down the intensity of chemical transformations [25,26,27]. The total number of microorganisms and *Pseudomonas* spp. is most frequently used to assess the microbiological quality of meat stored under aerobic conditions [10,14,15,18,28]. *Pseudomonas* spp. bacteria are considered specific microorganisms that spoil fresh meat during chilled storage. Poultry meat is particularly sensitive to the activity of these bacteria. Their impact involves the enzymatic acceleration of proteolysis, and oxidative and hydrolytic processes of fat tissues, generating unpleasant flavour and odour, improper colouring, and mucus [14,15,18,29,30]. The shelf life for consumption of poultry meat is most often determined on the basis of its sensory characteristics and the overall number of microorganisms, which should not exceed 7 log cfu/g [31]. Also, according to Gratta et al. [32], the termination of storage life of poultry meat is 7 log cfu/g for the total number of microorganisms and 7.3 log cfu/g for *Pseudomonas* bacteria, which are characteristic for spoiled poultry meat [15,16]. The shelf life of poultry meat depends mainly part of the initial microbiological load [15,33,34]. During the successive steps from poultry slaughtering to meat production, bacteria from the air and the environment can contaminate poultry meat. The skin of poultry carcasses and cuts is directly in contact with air and equipment surfaces, and is therefore easily contaminated. However, in fresh meat, bacteria are present on the surface rather than in the meat. Poultry meat is therefore exposed to a large number of bacteria that cause meat to spoil [17,18,35,36], and the initial level of bacterial contamination is greater than that of beef or pork [37]. An inappropriate storage temperature may be a factor that reduces the shelf life of cooled foodstuffs [6,16,38], especially when it undergoes fluctuations [39]. Traditional cooling temperatures in refrigeration equipment are usually between 0 °C and 7 °C, but often, consumers do not pay attention to the importance of the impact of storage temperature on the quality characteristics of meat without any treatment.

The purpose of this work was to assess the effect of temperature (2 °C and 6 °C) and storage time on the physicochemical, microbiological, and sensory characteristics of hen meat after the end of the laying period. The storage temperatures were chosen to account for the typical temperature range during refrigerated storage of poultry meat.

## 2. Materials and Methods

### 2.1. Research Material

The research material consisted of breast meat obtained from the carcasses of 56-week-old Rod Island Red (RIR) hens after the end of the laying period, of even body weights. RIRs are hens of dual-purpose breeds. The hens were on a certified ecological farm. During their entire period of use, the birds were raised according to the principles of ecological hen production, determined by the EU and national regulations [40,41,42]. The carcasses were subjected to a cooling process at a temperature of 4 °C. Maintaining the cooling chain, the carcasses were transported in isothermal containers to the laboratory. A simplified dissection according to the method of Ziołecki and Doruchowski [43] was conducted. During the dissection with a sterile knife, the breast meat (90 single units) was prepared, removing the external fat, which was used in further research. The evaluation of the material quality (day 0 of storage) was conducted on 10 single, randomly chosen meat breasts. The breast meat prepared for storage was divided into 2 experimental groups: the first group with a cool storage temperature of 2 °C; and the second group with a storage temperature of 6 °C. Each group included 40 randomly chosen meat breasts for the purpose of their further storage (for 2, 4, 6, 8 days) under stable temperature conditions in 2 refrigerators (FK v36/10 of Liebherr, Germany company). The samples (single meat breasts with a weight of 110 ± 5 g) were identified by weight, and were quickly and without delay transferred to separate marked containers for food storage. They were hand-wrapped in an air-permeable polyethylene foil, maintaining sterility. Each day, the samples were taken from 10 containers from the 2 refrigerators with the stable, monitored temperature conditions of 2 °C and 6 °C for further microbiological, physicochemical, and sensory evaluation.

### 2.2. Evaluation of the Physicochemical Characteristics of Meat

The pH level was measured with a digital pH meter-HI 99,163 (Hanna Instrument Company, Woonsocket, RI, USA) equipped with a combined electrode FC232 (Hanna Instrument Company, Woonsocket, RI, USA). Before measuring, the pH meter was calibrated using a two-point method towards the calibrated pH 4.01 and pH 7.01 buffers (Hanna Instrument Company, Salaj, Romania). The measures were conducted at the room temperature of 20–24 °C. The average pH value was determined based on 5 measurements of the same sample, and the procedures were the same for all samples. The surface colour of the cross section of the stored meat was determined with use of a colorimeter (CR-300; Minolta Camera, Osaka, Japan). Before measurement, the colorimeter was calibrated regarding the white standard to Y 94.2, *x* 31.63, *y* 33.30. A method of reflection was used with a standard lighting D65 observation angle of 2°. The product colour was shown as lightness (L*), redness (a*), and yellowness (b*), in accordance with the International Commission on Illumination (CIE) colour systems; the values were indicated from the average value of six randomly chosen readings. Tenderness was assessed on raw samples with the dimensions of (mm) 10 × 10 × 50, cut out parallelly to the course of muscle fibres. For measurement of the shear force (F max) use was made of a universal testing machine, Zwick/Roell BT1-FR1.OTH.D14 (Zwick CmbH&Co.KG., Ulm, Germany), with a Warner-Bratzler single-knife cutting system (one flat knife with a width of 1.2 mm with triangular incision at a 60° angle, of which the internal edge is also the working edge), with a head speed of 100 mm·min^−1^, and pretension force of 0.2 N. The cutting was conducted perpendicularly to the course of muscle fibres, and the temperature of the samples was 20 °C. The water holding capacity (WHC) was determined based on the amount of juice pressed out using the method of Grau and Hamm [44]. For this purpose, samples with a weight of 0.25–0.35 g were placed on tissue paper to press out the meat juice, and were loaded with a mass of 2 kg. After pressing out the juice, the samples were weighed again. Water absorption (%) was calculated from the following formula: M_1_ − M_2_/M_1_ × 100, where: M_1_—mass of test portion; and M_2_—mass of the test portion after squeezing out the juice. The drip loss (%) was determined on the basis of the loss in weight of the meat before (day 0) and after the storage period.

### 2.3. Microbiological Analysis

The study determined the total number of aerobic microorganisms, including Enterobacteriaceae, *Pseudomonas* spp., and *Salmonella* bacteria. In addition, typical microbial colonies were identified using MALDI-TOF MS Biotyper mass spectrometry by measuring unique bacterial proteins. Microbiological analysis was performed at 0, 2, 6, and 8 days of refrigerated storage at 2 °C and 6 °C. Each test subject was analysed in 3 independent replicates. Serial dilutions from 10^−1^ to 10^−5^ were made from the samples obtained. Total aerobic microbial counts to calculate the colony-forming units (cfu/g) were performed on a TSA medium (Triticasein Soy Lab-agar, Biomaxima, Lublin, Poland) according to PN-EN ISO 11133:2014-07 [45]. An endo Agar (Merck KGaA, Darmstadt, Germany), according to ISO 9308-1:2014 [46], used to determine Enterobacteriaceae bacteria, *Pseudomonas* spp. bacteria on a Pseudomonas Isolation Agar (Merck KGaA, Darmstadt, Germany) according to ISO 13720:2010 [47], and Salmonella bacteria on an SS medium (Biomaxima, Lublin, Poland) according to EN ISO 6579-1:2017 [48].

Qualitative analysis of microbial isolates was performed using the method described by Shell et al. [49] using MALDI-TOF mass spectrometry (Bruker Daltonics, Bremen, Germany). The generated spectra were analysed on a MALDI-TOF Microflex LT instrument (Bruker Daltonics, Bremen, Germany) using Flex Control 3.4 software. The probability of a correct identification was expressed by the instrument in point form. For scores between 2300 and 3000, a reliable identification of microorganisms to the species level was obtained; for 2000–2299, reliable designation to the genus level and probable identification to the species level were achieved; scores between 1700 and 1999 indicated a likely identification result to the genus level; and scores below and/or equal to 1699 produced an unreliable identification result [50]. The presented results fall within the range of 2000–3000. The MaldI-TOF MS method (matrix-assisted laser desorption/ionisation time-of-flight mass spectrometry) method is based on the analysis of the protein profile of the organism [51]. This method has found its special place in meat microbiology, as a fast and inexpensive method, additionally characterised by high accuracy in the identification of bacteria. The identification of microorganisms is primarily based on the detection of ribosomal proteins, but also mitochondrial proteins that can be isolated [52,53,54,55].

### 2.4. Sensory Evaluation

Evaluation of the sensory parameters of samples in cold storage was conducted by a 10-person assessment team with tested sensory sensitivity and at least 4 years of experience in evaluation using a scaling method. The panellists were trained in sensory analysis according to ISO 8586-2 and familiarised themselves with the evaluation form prior to participating in the study [56].

The study made use of a specially prepared evaluation form.

A 5-point hedonic scale was used. The attributes subject to evaluation were: intensity and desirability of odour (5—very strong, typical; 4—strong, typical; 3—weak, unnoticeable, typical; 2—slightly changed, not strong, 1—changed, spoiled); colour of cross section and external colour (5—even, typical; 4—desired, less even, typical; 3—moderately desired, uneven, changed in places; 2—slightly undesired, changed in places, infiltrations, yellow; 1—very undesired, changed in places, yellow or green); texture (5—resilient muscle tissue, dense; 4—averagely resilient, deformation evens out after pressing; 3—muscle tissue after finger pressure remains deformed; 2—muscle tissue loosened, flattens after pressing; 1—muscle tissue loosened after pressing, easily separates); general appearance (5—no objections, surface moist, typical; 4—desired, surface slightly dried; 3—moderately desired, surface dry or slightly moist; 2—undesired, surface slimy, slightly sticky, colour changed in places; 1—very undesired, surface sticky from mucus). To ensure a proper assessment, the samples were coded and presented to the evaluators in white containers. The tests were carried out in appropriately prepared rooms free of foreign odours, at a temperature of 20 °C and with lighting eliminating any distracting factors, according to the relevant standards [57].

### 2.5. Statistical Analysis

The results obtained were statistically analysed using the analysis of variance (ANOVA) and Statistica 13.3 software package [58]. The research results are tabulated as the mean values, standard deviation, and range. The collected data were verified for normality using the Kolmogorov–Smirnov test. The impact of the effect of temperature (2 °C and 6 °C) and storage time (for 2, 4, 6, and 8 days) on the physicochemical and microbiological characteristics of the hen meat after the end of the laying period was evaluated with a Tukey’s post hoc tests. The sensory evaluation was characteristics using non-parametric Kruksal–Wallis tests. Differences were considered significant at *p* < 0.05.

## 3. Results and Discussion

During storage, the physical–chemical characteristics of meat may undergo changes, including in pH, water absorption, colour, and tenderness. In our study, the acidity (pH) of raw hen breast meat on the first day after slaughter remained at the level of 5.62 ± 0.02 (Table 1). With increased storage time, both at a temperature of 2 °C and at a temperature of 6 °C, the degree of acidity of the meat increased. The increase in pH level of the meat during storage at a temperature of 6 °C was greater than at a temperature of 2 °C, and on the 8th day of storage reached the level of 6.89 ± 0.10. The production of lactic acid bacteria and the accumulation of alkaline components produced by psychrotrophic bacteria and the autolytic activity of the autochthonous enzymes may be the main reason for the change in pH during storage [59]. The increase in pH level of the meat during storage may be caused by the activity of microorganisms developing in the meat, which have the ability to produce enzymes catalysing the process of deamination of amino acids, which, consequently, causes the formation of nitrogenous bases and ammonia [26,59,60].

An increase in pH levels during the storage of poultry meat was also demonstrated by Surmei et al. [61], Ruíz-Cruz et al. [62], Nikmanesh et al. [59], Saleh et al. [25], and Katiyo et al. [12]. In the research of Sujiwo et al. [7], concerning the storage of broiler chicken meat at a temperature of 4 °C, no significant changes in the pH level were noted until the 9th day of storage. In our study, a high pH level (6.89) of meat on the 8th day of storage at a temperature of 6 °C was accompanied by the loss of suitability for consumption, which is concurrent with the results of Surmei and Usturoi [63], who stated that poultry meat is considered to be of very good quality with a pH not exceeding 6.2, whereas at a pH higher than 6.7, meat becomes unsuitable for consumption. In their study, Katiyo et al. [12] stated that changes in the pH level of meat have a significant influence on other meat characteristics, including colour and water absorption.

Our research demonstrated that the storage temperature had a considerable influence on the water holding capacity (WHC). Storage temperature and time, and microbiological growth are the main factors that influence the water retention ability of myofibrils in meat during storage under cold conditions. A significant decrease in water holding capacity was shown at the lower storage temperature. At a lower storage temperature, a significant fall in water absorption was observed on the 8th day of storage (Table 1). The research results obtained are consistent with the results of Sinhamahapatra et al. [64], Aziman et al. [65], and Hussein et al. [21] for the water absorption of broiler chicken meat during storage at a temperature of 4 °C. Most likely, the reduction in water contained in the meat with the passage of storage time is a result of greater leakage from mature meat, which in turn can increase the relative share of raw protein in stored meat [21]. The leakage of the cooling meat stored at temperatures of 2 °C and 6 °C increased along with the storage time at both temperatures of 2 °C and 6 °C (Table 1). The losses of cooled meat depend on the pH of the meat [66]. Poultry meat with a low pH has been associated with low water holding capacity (WHC), which results in increased cook loss, drip loss, and shelf life, and decreased tenderness. Similar results were noted in the research of Ruíz-Cruz et al. [62], and Chmiel and Słowiński [6], as well as Kondratowicz et al. [30].

Measuring the shear force is the most effective method of evaluating the tenderness of meat [62], including a raw meat. Our study indicated that the tenderness of the meat stored at a temperature of 6 °C, measured as a value of shear force, increased significantly (*p* < 0.05) from the 4th day of storage, while increased tenderness of the meat stored at a temperature of 2 °C was not noted until the 8th day of storage (Table 1). A reduction in the shear force during storage was observed in the studies of Sujiwo et al. [7] and Gratta et al. [32]. In the study by Gratta et al. [32], the test material was also raw meat. In the work of Ruíz-Cruz et al. [62], the shear force of broiler chicken fillets was reduced over the first 4 days of cool storage and increased in the next days. The tenderness of meat depends on myofibril proteolysis, which helps maintain the integrity of muscle fibre [63]. In the opinion of Kruk et al. [67], protein degradation in meat may be caused by bacterial or enzymatic processes occurring in meat in cold storage.

In our research it was indicated that along with the storage time and lightness (L*) of colour, storage at a temperature of 2 °C did not present significant changes. However, while hen meat after the laying period stored at a temperature of 6 °C on the 4th, 6th, and 8th day of storage was characterised by a darker colour (lower indicator of lightness L*) then on the first and second days of storage. The colour of the meat changed at 6 °C, where a higher proportion of red (parameter a*) and yellow (parameter b*) was shown compared to the meat stored at 2 °C (Table 1), regardless of storage time. The darkening of the chicken meats may be due to the reduction in the oxygen level in the surface tissue caused by microbial growth. This oxygen level reduction promotes the oxidation or denaturation of myoglobin and the formation of deoxymyoglobin, resulting in the degradation of the red colour within chicken meat [60]. At a temperature of 6 °C, the colour saturations of red (a*) and yellow (b*) also underwent changes. The increased colour saturation of yellow in the meat stored at the temperature of 6 °C was greater than at the temperature of 2 °C (Table 1). The results of the colour darkening during the 7-day storage period were obtained by Aziman et al. [65]. Changes in lightness during the storage period were also noted by Gratta et al. [32].

An approximative indicator of the microbiological quality of refrigerated meat is the overall number of microorganisms. This indicator assesses the total concentration of microorganisms in a sample, and helps determine the meat’s level of hygiene and remaining shelf life [10,32,68]. However, *Pseudomonas* spp. bacteria are generally considered to be the microorganisms causing the spoiling of poultry meat stored in aerobic conditions [14,15,18,21,32,69]. Enterobacteriaceae are one of the potential bacterial spoilage groups in poultry meat [15,20,70]. However, the involvement of these bacteria and their role in poultry meat spoilage has not been fully investigated [71].

The research we conducted demonstrated that at a temperature of 2 °C, the overall number of bacteria of the family *Enterobacteriaceae* and the number of *Pseudomonas* spp. bacteria in the meat gradually increased, reaching the respective levels 4.64 log cfu/g, 4.16 log cfu/g, and 4.48 log cfu/g on the 8th day of cold storage (Table 2). In the meat stored at the temperature of 6 °C, the tempo of bacterial growth in all the assessment periods was significantly greater than at the temperature of 2 °C (Table 2). On the 6th day of storage at 6 °C, the overall number of bacteria in the meat exceeded 7 log cfu/g, considered to be the limit of microbiological safety [8]. On the 8th day of storage, microbiological evaluation of the meat stored at the temperature of 6 °C was ceased, as the sensory characteristics of the meat indicated its spoilage (Table 3) and loss of suitability for consumption.

The increase in the overall number of bacteria in poultry meat stored in cooling conditions at a temperature of 4 °C was noted by Sujiwo et al. [7] and by Vergara-Figueroa et al. [20], and at the storage temperatures of 5 °C and 10 °C, as indicated by Ntzimani et al. [17]. Gratta et al. [32] demonstrated that both the overall number of microorganisms and of the *Pseudomonas* spp. bacteria increased along with the storage time of the poultry meat, normal and with myopathy, at a temperature of 4 °C, as well as Dourou et al. [18] at temperatures 0–5 °C in a refrigerator. The growth of Enterobacteriaceae in poultry meat in cold storage was indicated by Rouger et al. [15] and Vergara-Figueroa et al. [20]. Our research has demonstrated that in hen meat after the laying period, the presence of disease-causing bacteria (*Salmonella* spp.) was not noted, which reflects the good state of the health of hens from which the meat was obtained, as well as good sanitary conditions during the raising and slaughter of hens and proper storage conditions [3].

**Figure 1:**
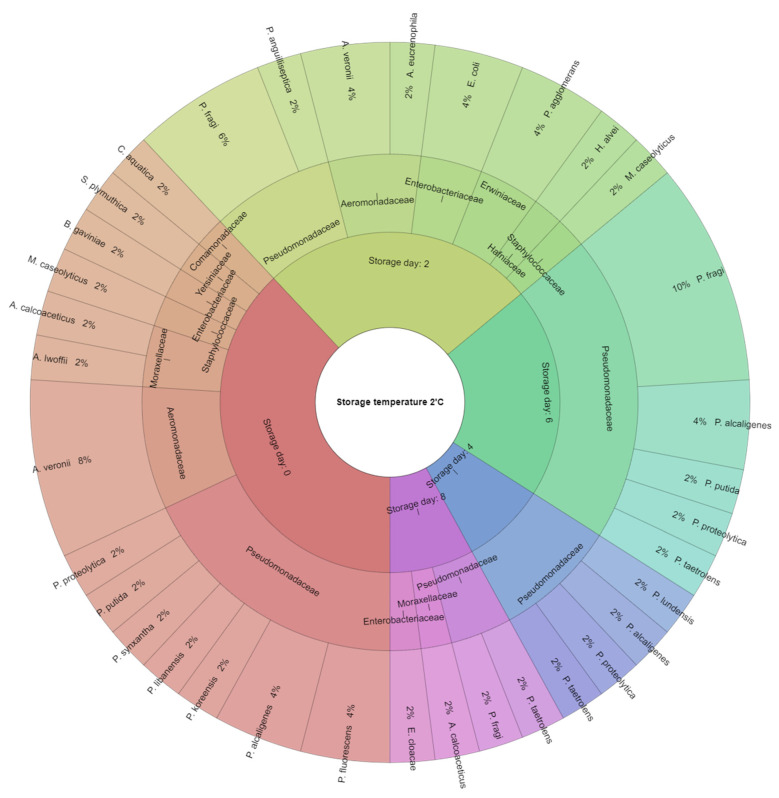
Isolated species of the bacteria from hen meat after the laying period storage at 2 °C.

The study was conducted with the use of a MALDI-TOF MS Biotyper, allowing for an identification of bacteria in the meat stored at the temperatures of 2 °C and 6 °C. The results obtained in the current study have indicated that the initial microflora of hen meat after the laying period is consistent with published data for poultry meat [72,73,74]. From the test material obtained, 106 bacteria were isolated and correctly identified (score above 2000), of which the vast majority, 94%, were Gram-negative bacteria. The bacteria were clustered between nine families: Pseudomonadaceae (27%), Enterobacteriaceae (26%), Aeromonadaceae (18%), Staphylococcaceae (6%) and Moraxellaceae, Hafniaceae, Erwiniaceae, Comamonadaceae, and Yersiniaceae (less than 5%) (Figure 1). Based on the German Rules for Biological Agents #446, it was found that of the correctly identified bacteria, 34% were categorised as risk factor 1 (Biological agents that are unlikely to cause disease in an individual), while the remaining 66% were classified as risk factor 2 (biological agents that are likely to cause disease in humans and pose a risk to workers; spread in the community is unlikely; and effective prevention or treatment is usually possible). On day 0 of storage (24 h after cutting), bacteria belonging to four families were identified: Pseudomonadaceae (47%), Aeromonadaceae (21%), and Moraxellaceae (11%), as well as Staphylococcaceae, Enterobacteriaceae, Yersiniaceae, and Comamonadaceae (5% each). In the Pseudomonadaceae family, seven bacterial species were isolated: *Pseudomonas fluorescens* (11%), *Pseudomonas alcaligenes* (11%), *Pseudomonas koreensis* (5%), *Pseudomonas libanensis* (5%), *Pseudomonas synxantha* (5%), *Pseudomonas putida* (5%), and *Pseudomonas proteolytica* (5%). The Aeromonadaceae family was represented by *Aeromonas veronii* (21%), while the Moraxellaceae family by *Acinetobacter lwoffii* and *Acinetobacter calcoaceticus* (5% each). Among the Staphylococcaceae, the bacteria *Macrococcus caseolyticus* (5%) was isolated; in the Enterobacteriaceae family, the bacterium *Buttiauxella gaviniae* (5%) was isolated; among the Yersiniaceae, the bacterium *Serratia plymuthica* (5%) was isolated; and from the Comamonadaceae family, the bacterium *Comamonas aquatic* was identified. Bacteria from the family Moraxellaceae, Pseudomonadaceae, and members of the Vibrionaceae family were found in the meat of egg-laying poultry. *Pseudomonas* was found to be the predominant psychotropic spoilage-causing agent in meat [75,76,77,78,79]. On the 2nd day, the meat stored at 2 °C also had the highest percentage of bacteria from the Pseudomonadaceae family (31%), followed by Aeromonadaceae (23%), Enterobacteriaceae and Erwiniaceae (15% each), and the Hafniaceae and Staphylococcaceae families (8% each). Among the bacteria of the Pseudomonadaceae family, *Pseudomonas fragi* (23%) and *Pseudomonas anguilliseptica* (8%) were identified. *A. veronii* (15%) and *Aeromonas eucrenophila* (8%) were the bacteria correctly identified in the family Aeromonadaceae. The Enterobacteriaceae family was represented by *Escherichia coli* bacteria (15%), and Erwiniaceae by *Pantoea agglomerans* bacteria (15%). *Hafnia alvei* and *Macrococcus caseolyticus* bacteria (8% each) from the Hafniaceae and Staphylococcaceae families, respectively, were also identified.

On the 4th day of storage at 2 °C, all identified bacteria belonged to the *Pseudomonadaceae* family: *P. lundensis*, *P. alcaligenes*, *P. proteolytica*, and *P. teatrolens* in equal proportion. Additionally, on day six, bacteria from only one bacterial family were identified. *Pseudomonadaceae* was represented by *P. fragi* (50%), *P. alcaligenes* (20%), *P. putida* (10%), *P. proteolytica* (10%), and *P. teatrolens* (10%) (Figure 1). On the last eight days of storage of the meat sample at 2 °C, bacteria from the Pseudomonadaceae (50%), Moraxellaceae (25%), and Enterobacteriaceae (25%) families were identified. Among the bacteria of the Pseudomonadaceae family, *P. fragi* and *P. teatrolens* were identified (25% each), while for the Moraxellaceae family, *A. calcoaceticus* bacteria (25%) was identified, and among Enterobacteriaceae, *Enterobacter cloacae* bacteria (25%) was isolated.

**Figure 2:**
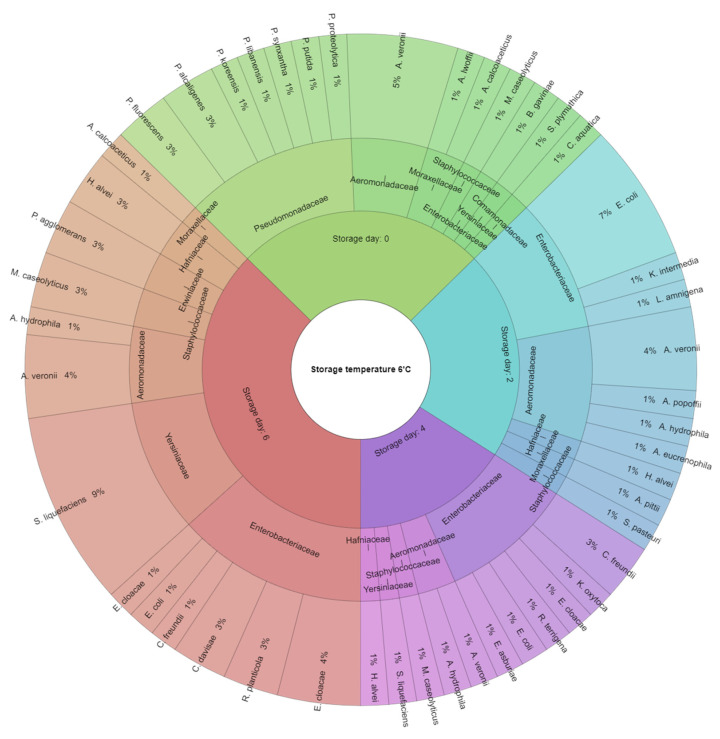
Isolated species of the bacteria from hen meat after the laying period storage at 6 °C.

On the 2nd day of meat storage at 6 °C, bacteria belonging to five different families were identified: Enterobacteriaceae (44%), Aeromonadaceae (38%), and Hafniaceae, Moraxellaceae, and Staphylococcaceae (6% each). In the Enterobacteriaceae family, the following were identified: *E. coli* (31%), *Kluyvera intermedia* (6%), and *L. amnigena* (6%). Among Aeromonadaceae, the following bacteria were isolated: *A. veronii* (19%) and *Aeromonas popoffii*, *Aeromonas hydrophila*, and *Aeromonas eucrenophila* (6% each). In addition, from the Hafniaceae family, the bacterium *H. alvei* (6%) was found; from the *Moraxellaceae* family, the bacterium *Acinetobacter pittii* (6%) was isolated; and from the *Staphylococcaceae* family, bacteria *S. pasteuri* (6%) were identified (Figure 2).

On the 4th day of storage at 6 °C, bacteria belonging to the following families were identified: Enterobacteriaceae (58%) and Aeromonadaceae (17%), as well as Staphylococcaceae, Hafniaceae, and Yersiniaceae (8% each). In the Enterobacteriaceae family, the bacteria identified were *Citrobacter freundii* (17%), as well as *Klebsiella oxytoca*, *E. cloacae*, *E. coli*, *Raoultella terrigena*, and *Enterobacter asburiae* (8% each); and in the Aeromonadaceae family, the bacteria *A. hydrophila* and *A. veronii* (8% each) were isolatead. In the case of bacteria from the families Staphylococcaceae, Hafniaceae, and Yersiniaceae, one genus each was identified, and these were *M. caseolyticus*, *H. Alvei*, and *Serratia liquefaciens* (8% each), respectively.

On the 6th day of meat storage at 6 °C, bacteria belonging to seven families were identified: Enterobacteriaceae (36%), Yersiniaceae (25%), and Aeromonaidaceae (14%), as well as Staphylococcaceae, Erwiniaceae, Hafniiaceae (7% each), and Moraxelliaceae (4%). In the Enterobacteriaceae family, the following bacteria were identified: *I. cloacae* (14%), *Raoultella planticola* (7%), *Cedecea* daivisae (7%), *C. freunidii* (4%), and *E. coli* (4%). In the family *Yersiniaceae*, the bacteria *S. liquefaciens* (25%) were identified; in the family Aeromonadiaceae, the bacteria *A. veronii* (11%) and *A. hydrophila* (4%) were isolated. On the other hand, from the family Staphylococcaceae, Erwiniaceae, Hafniaceae, and Moraxellaceae, the bacteria identified were *M. caseolyticus*, *P. agglomerans*, *H. alvei* (7% each), and *Acinetobacter calcoaceticus* (4%), respectively.

The composition of the bacterial microflora in the meat changed during storage, as confirmed by culture tests and identification via the MALDI-TOF MS Biotyper. The main microflora, regardless of the temperature used, were psychrophilic bacteria, which are characteristic and able to grow under refrigerated conditions. However, it was observed that during storage at 6 °C, the profile of the identified bacteria changed, with the majority of unfavourable microflora appearing, indicative of progressive spoilage processes. These bacteria may include, among others, *Aeromonas* spp., *Alcaligenes* spp., *Klebsiella* spp., and *Yersinia* spp. [20,75,80].

The effects of cool storage conditions on the sensory conditions of poultry meat were presented in the research of Chmiel and Słowiński [6], Ruíz-Cruz et al. [62], Yimenu et al. [36], Sujiwo et al. [7], and Kondratowicz et al. [30], as well as Garavito et al. [16]. In the study we conducted, over the storage time, all of the tested sensory characteristics of meat deteriorated, i.e., the intensity and desirability of odour, colour, texture, and general appearance (Table 3). The most obvious changes involved the odour of meat stored at a temperature of 6 °C. The intensity and desirability of the odour were reduced over the storage time.

Also, in the research of Katiyo et al. [12], the odour of chicken meat deteriorated more quickly than the colour or general appearance, and was strongly correlated with the growth in the number of microorganisms, which led the authors to state that the odour of raw meat may be a more reliable signal of rotting brought on by the development of microorganisms than the meat’s appearance. The sensory quality of the breast meat of broiler chickens packed [36,81] and coated in an antibacterial coating [16] was reduced at a pace dependent on the temperature and increased storage time. In our research, the meat stored at a temperature of 2 °C was characterised by an acceptable, though not the highest, sensory quality until the 8th day of storage. On the other hand, the sensory characteristics of the meat stored at 6 °C on the 8th day of storage indicated its spoilage and loss of suitability for consumption (Table 3). According to Katiyo et al. [12], unpacked broiler chicken meat stored under aerobic conditions longer than 7 days was characterised by detrimental sensory characteristics and a total number of microorganisms higher than 8 log cfu/g.

## 4. Conclusions

The meat of hens of dual-purpose breeds after the laying period is over is now most often a waste product. In recent years, however, there has been growing interest in the possibility of using meat from the hens of such breeds. The results obtained from the evaluation of sensory, physicochemical, and microbiological characteristics showed that hen meat after the laying period stored under refrigerated conditions at 6 °C retains its shelf life only for up to 4 days, while storage at 2 °C retains shelf life up to 8 days of storage. The results obtained allow us to conclude that lowering the refrigerator temperature from 6 °C to 2 °C preserves the sensory characteristics and microbiological safety of post-lay hen meat at an acceptable level for longer, and therefore extends its shelf life.

The study provides new insights in terms of monitoring the breast muscle of laying hens after laying at different storage temperatures during different days, and gives an overview of how different bacterial species develop at different storage temperatures. The study provides new insights for practitioners in this field and identifies exactly which microorganisms develop during storage.

## Figures and Tables

**Table 1:** Physicochemical characteristics of hen meat after the laying period depending on the temperature and period of time in cool storage (means ± standard deviation).

Tested Parameter	0 Day	Storage Temp. (°C)	Refrigerated Storage Time (Days)				
			2	4	6	8	*p*-Value
pH	5.62 ^a^ ± 0.02	2	5.66 ^a^ ± 0.03	5.77 ^b^ ± 0.03	5.82 ^b^ ±0.02	5.85 ^b^ ± 0.02	<0.001
		6	5.67 ^a^ ± 0.04	6.20 ^b^ ± 0.24	6.25 ^b^ ± 0.08	6.89 ^c^ ± 0.10	<0.001
		*p*-value **	0.491	<0.001	<0.001	<0.001	
Drip loss (%)	-	2	0.93 ^a^ ± 0.15	1.39 ^a^ ± 0.15	2.01 ^b^ ± 0.12	2.32 ^b^ ± 0.10	<0.001
		6	1.07 ^a^ ± 0.10	2.39 ^b^ ± 0.25	2.92 ^c^ ± 0.26	3.15 ^c^ ± 0.23	<0.001
		*p*-value**	0.026	<0.001	<0.001	<0.001	
WHC (%)	24.39 ^b^ ± 2.34	2	31.81 ^a^ ± 2.58	25.00 ^b^ ± 2.61	23.33 ^b^ ± 2.80	28.53 ^b^ ± 3.10	0.041
		6	31.32 ± 2.32	30.41 ± 2.56	32.25 ± 2.40	32.041.12	0.066
		*p*-value **	0.232	<0.001	<0.001	<0.001	
Colour: L*—lightness	57.02 ^a^ ± 3.20	2	56.17 ^a^ ± 1.78	54.62 ^b^ ± 1.89	54.50 ^b^ ± 1.83	53.42 ^b^ ± 1.88	<0.001
		6	56.17 ^a^ ± 1.78	49.27 ^b^ ± 1.33	49.30 ^b^ ± 1.13	45.60 ^b^ ± 1.79	<0.001
		*p*-value **	0.023	<0.001	<0.001	<0.001	
a*—redness	3.69 ^a^ ± 0.62	2	3.73 ^a^ ± 0.66	3.78 ^a^ ± 0.28	3.90 ^a^ ± 0.36	4.22 ^b^ ± 0.45	0.044
		6	3.78 ^a^ ± 0.27	4.20 ^b^ ± 0.70	3.98 ^ab^ ± 0.25	4.28 ^c^ ± 0.39	0.003
		*p*-value **	0.051	0.030	0.535	0.902	
b*—yellowness	6.78 ^a^ ± 0.02	2	9.76 ^b^ ± 0.76	11.58 ^c^ ± 1.26	11.49 ^c^ ± 0.49	11.98 ^c^ ± 1.19	<0.001
		6	10.09 ^b^ ± 1.36	13.16 ^d^ ± 1.30	14.02 ^d^ ± 0.89	13.41 ^d^ ± 0.85	<0.001
		*p*-value **	0.504	0.004	<0.001	0.020	
Shear force (N)	31.20 ^a^ ± 4.50	2	31.00 ^a^ ± 2.70	30.25 ^a^ ±3.40	28.90 ^a^ ± 3.62	25.60 ^b^ ± 3.80	0.038
		6	30.68 ^a^ ± 4.50	25.70 ^b^ ± 2.80	20.84 ^c^ ± 3.20	19.90 ^c^ ± 2.80	0.002
		*p*-value **	0.502	0.032	<0.001	<0.001	

** *p*-value, the level of significance when estimating the differences between the means of the parameters tested at storage temperatures of 2 and 6 °C; ^a,b,c,d^—marked with different letters in the rows differ.

**Table 2:** The microbiological quality of hen meat stored at temperatures of 2° and 6 °C (log cfu/g) (means ± standard deviation and range).

Parameter	0 Day	Storage Temp. (°C)	Refrigerated Storage Time (Days)				
			2	4	6	8	*p*-Value
Total microorganism count	3.48 ^a^ ± 0.39	2	3.50 ^a^ ± 0.05	3.72 ^a^ ± 0.44	3.80 ^a^ ± 0.54	4.64 ^b^ ± 0.38	<0.001
			3.43–3.57	3.30–4.30	3.30–4.43	4.34–5.34	
		6	4.11 ^a^ ± 0.45	6.29 ^b^ ± 0.15	7.07 ^c^ ± 0.37	nt	<0.001
	2.85–3.87		3.63–4.66	6.13–6.47	6.62–7.62		
		*p*-value **	0.007	<0.001	<0.001		
Family Enterobacteriaceae	<2	2	3.61 ^a^ ± 0.13	3.57 ^a^ ± 0.41	3.75 ^a^ ± 0.13	4.16 ^b^ ± 0.32	0.005
			3.40–3.73	3.14–3.99	3.59–3.91	3.70–4.43	
		6	3.70 ^a^ ± 0.10	5.70 ^b^ ± 0.54	6.76 ^c^ ± 0.09	nt	<0.001
			3.53–3.80	5.09–6.24	6.62–6.88		
		*p*-value **	0.248	<0.001	<0.001		
*Pseudomonas* spp.	2.87 ^a^ ± 0.35	2	2.85 ^a^ ± 0.77	3.69 ^b^ ± 0.13	3.70 ^b^ ± 0.14	4.48 ^c^ ± 0.25	<0.001
			2.20–3.32	3.59–3.83	3.51–3.91	4.00–4.66	
		6	3.70 ^a^ ± 0.04	6.32 ^b^ ± 0.12	7.11 ^c^ ± 0.23	nt	<0.001
	2.41–3.32		3.62–3.73	6.18–6.51	6.73–7.38		
		*p*-value **	0.023	0.020	0.007		
*Salmonella* spp.	nd	2	nd	nd	nd	nd	
		6	nd	nd	nd	nd	

nd—not detected; nt—not tested; ** *p*-value, the level of significance when estimating the differences between the means of the parameters tested at storage temperatures of 2 and 6 °C; ^a,b,c^—marked with different letters in the rows differ.

**Table 3:** Evaluation of sensory characteristics of hen meat stored at temperatures of 2 °C and 6 °C (means ± standard deviation).

Parameter	0 Day	Storage Temp. (°C)	Refrigerated Storage Time (Days)			
			2	4	6	8
Intensity and desirability of the odour	4.90 ^a^ ± 0.30	2	4.62 ^a^ ± 0.30	^A^ 4.10 ^ab^ ± 0.30	^A^ 3.90 ^ab^ ± 0.32	^A^ 3.30 ^b^ ± 0.52
		6	4.10 ^a^ ± 0.42	^B^ 2.60 ^b^ ± 0.63	^B^ 1.80 ^c^ ± 0.42	^B^ 1.00 ^c^ ± 0.48
Outside colour	4.80 ^a^ ± 0.41	2	4.60 ^a^ ± 0.50	^A^ 3.70 ^ab^ ± 0.51	^A^ 3.80 ^ab^ ± 0.42	^A^ 3.31 ^b^ ± 0.56
		6	4.30 ^a^ ± 0.60	^B^ 2.50 ^b^ ± 0.42	^B^ 2.60 ^b^ ± 0.69	^B^ 1.40 ^c^ ± 0.51
Consistency	4.65 ^a^ ± 0.50	2	4.70 ^a^ ± 0.48	^A^ 3.90 ^b^ ± 0.30	^A^ 3.50 ^b^ ± 0.52	^A^ 3.00 ^c^ ± 0.46
		6	3.50 ^a^ ± 0.36	^B^ 2.20 ^ab^ ± 0.48	^B^ 1.40 ^c^ ± 0.52	^B^ 1.30 ^c^ ± 0.48
General appearance	4.85 ^a^ ± 0.36	2	4.60 ^a^ ± 0.52	^A^ 3.90 ^ab^ ± 0.44	^A^ 3.70 ^ab^ ± 0.48	^A^ 3.00 ^b^ ± 0.52
		6	3.70^a^ ± 0.52	^B^ 2.30 ^b^ ± 0.52	^B^ 1.60 ^bc^ ± 0.52	^B^ 1.50 ^c^ ± 0.52

^A,B^—marked with different letters in the columns differ at *p* ≤ 0.05; ^a,b,c^—marked with different letters in the rows differ at *p* ≤ 0.05.

## Data Availability

The data used to support the findings of this study can be made available by the corresponding author upon request.
